# Comparison of 3D ankle kinematics between minimal inertial measurement units configuration and optical motion capture system under diverse walking conditions

**DOI:** 10.1038/s41598-026-39161-8

**Published:** 2026-02-11

**Authors:** Jongsu Kim, Lingchao Xie, Sanghyun Cho

**Affiliations:** 1 Department of Physical Therapy, Yonsei University, Wonju, 26493 South Korea; 2 Department of Neurosurgery, Taizhou University, Taizhou, 317500 People’s Republic of China

**Keywords:** Optical motion capture system, Inertial measurement units, Ankle kinematics, Gait analysis, Diverse walking conditions, Validity and repeatability, Anatomy, Engineering, Health care, Medical research

## Abstract

Accurate assessment of three-dimensional ankle kinematics is essential for advancing biomechanical analyses and informing potential clinical applications. While optical motion capture systems (OMCs) are considered the gold standard, their high cost and limited portability restrict their use outside the laboratory. Inertial measurement units (IMUs) offer a more practical alternative; however, concerns remain regarding their validity and reliability in diverse walking conditions. This study evaluated the agreement and repeatability of IMU-derived ankle kinematics relative to OMCs across three walking surfaces: level ground, inward wedge, and outward wedge. Ankle movements in the sagittal, frontal, and transverse planes were analyzed using a minimal sensor configuration. Moderate to high agreement and consistent repeatability were observed in the sagittal and transverse planes, particularly during level and inward wedge floor walking. Conversely, the frontal plane demonstrated limited agreement under wedged conditions, likely because of complex multisegmental foot dynamics and reference frame misalignment from static calibration. Despite these limitations, consistent repeatability across conditions supports the use of IMUs for tracking ankle kinematics outside the laboratory. These findings suggest the preliminary applicability of IMUs as a functional tool for interpreting ankle kinematics outside the laboratory.

## Introduction

 Three-dimensional (3D) ankle kinematics are crucial in gait analysis and offer valuable insights for clinical rehabilitation, injury prevention, and biomechanical assessment. Accurate measurement of joint motion enables monitoring of functional recovery by identifying injury risk factors and diagnosing gait abnormalities^1–3^. Therefore, a comprehensive assessment of 3D ankle kinematics is essential for both clinical and research applications. Optical motion capture systems (OMCs) are widely regarded as a gold standard for kinematic analysis because of their high accuracy, despite occasional challenges such as soft tissue artefact and marker misplacement^4^. Additionally, their practical application is limited by the need for numerous markers, high costs, complex data processing, and poor applicability outside laboratory settings.

Accordingly, the interest in alternative methods that offer flexibility and practicality has been increasing. Inertial measurement units (IMUs) have emerged as promising substitutes to OMCs, which provide advantages such as portability, cost-effectiveness, and ease of use^5,6^. Unlike OMCs, IMUs require only a single sensor per segment to capture 3D joint kinematics, thereby enabling a significantly simplified setup^7^. This not only lowers costs but also facilitates data processing. Despite these advantages, several challenges hinder the extensive adoption of IMUs.

Among the key challenges identified in prior studies, methodological limitations are particularly significant. Substantial research efforts have focused on sagittal plane kinematics, despite the relevance of frontal and transverse planes for understanding functional ankle movement^3,7^. Additionally, the effects of diverse walking conditions have often been overlooked, although such conditions are crucial for evaluating the applicability of IMUs beyond controlled environments^8,9^. Furthermore, while a single sensor per segment is theoretically sufficient to measure joint kinematics, many studies have employed multiple sensors to capture several joints simultaneously^10,11^. These limitations collectively reduce the practical utility of IMU-based assessments, particularly in real-world settings.

Therefore, a comprehensive evaluation of the IMU-based assessments is necessary to ensure their generalizability and practical relevance^12–14^. Analyzing ankle kinematics across all three anatomical planes enables the detection of subtle movement variations that reflect the ankle’s multidimensional motions. Measuring performance under varied conditions introduces significant environmental perturbations, which offer insights into sensor sensitivity to external factors. Furthermore, focusing on a single joint while simplifying the sensor setup could improve the efficiency of IMU applications. These methodological refinements support the broader use of IMUs beyond laboratory environments.

This study evaluated the reliability and validity of 3D ankle kinematics measured by IMUs and OMCs under varied walking conditions by using a minimal sensor setup. We hypothesize that IMU-derived measurements would demonstrate strong agreement with those from OMCs across all three anatomical planes. To test this hypothesis, we quantified the statistical relationship between the two measurement systems by using waveform similarity, performed time-series comparisons, and conducted repeatability analyses. Confirmation of this hypothesis would support the adoption of minimal sensor configurations for accurate ankle kinematic assessments.

## Method

### Participants

Twelve healthy young adults (6 males, 6 females; age: 19–33 years; height: 1.58–1.83 m; body mass: 50.6–92.8 kg) participated in this study. The inclusion criteria required the absence of gait abnormalities or lower-extremity musculoskeletal disorders within the past 6 months. The sample size was determined through a power analysis to ensure sufficient statistical robustness for statistical parametric mapping^15^. Ethical approval was obtained from the Yonsei University Institutional Review Board (IRB No. 1041849-202405-BM-109-03), and all participants provided written informed consent before participation.

### Protocol

**Fig. 1:**
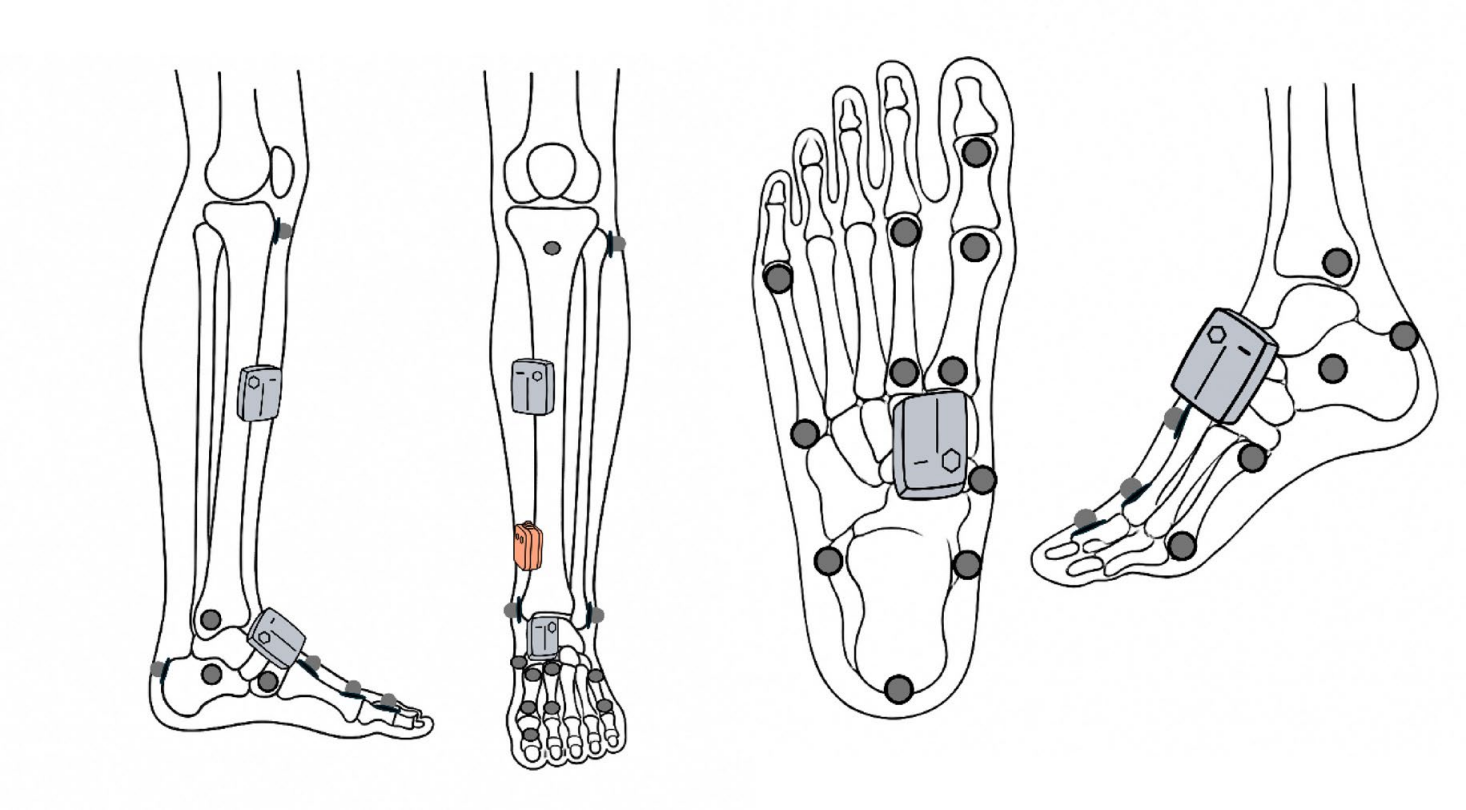
Marker setup and sensor placement. Optical markers (gray circles) were attached according to the Institute of Rizzoli (IOR) foot model to enable 3D ankle kinematic reconstruction with the optical motion capture system (OMCs). Inertial measurement units (gray rectangles) were affixed to the shank and the dorsal foot to record segment orientations. Additionally, gyroscope (orange rectangle) was mounted on the shank for gait event detection.

A Vicon motion capture system (Vicon Motion Systems Ltd., Oxford, UK) equipped with eight cameras was used as OMCs. Optical markers were placed based on the IOR foot model, which provides anatomically consistent segment alignment for ankle kinematics assessment (Fig. 1). This model is advantageous because of its coordinate system definitions, which are aligned with anatomical bone structures and correspond directly to the anatomical planes of motion^2^. Moreover, its marker-based angle computation is functionally comparable to the IMU algorithm, wherein each segment is represented by a sensor to estimate joint angles^16^.

Two IMU sensors (Ultium Motion, Noraxon, AZ, USA) were attached to the shank and dorsal foot following the manufacturer’s guidelines to define segment axes and enable 3D ankle kinematic estimations^17^. While the IOR foot model defines axes based on anatomical landmarks, the IMU axes were functionally aligned to approximate anatomical axes. This configuration represents the minimal setup required to calculate joint angles. The simplified setup reduces system complexity and user burden, thereby enhancing its practicality for real-world applications.

The participants performed static trials to establish OMCs baseline data, with a neutral standing position used to define the reference frames for each marker^18^. For the IMUs, static calibration was performed on a level surface before the start of each trial in all walking conditions. During calibration the accelerometer provided the gravity vector, which was used to align the IMU vertical axis with the anatomical vertical. The magnetometer was then used to determine the direction relative to the laboratory coordinate system, ensuring that the IMU anterior axis was aligned with the subject’s forward orientation. The shank and foot IMU axes were thus functionally aligned to approximate the anatomical axes of each segment, providing local reference frames for subsequent joint angle computation. Drift was minimized using the extended Kalman filter, which integrates gyroscope signals with accelerometer- and magnetometer-based corrections to stabilize orientation over time^19,20^.

**Fig. 2:**
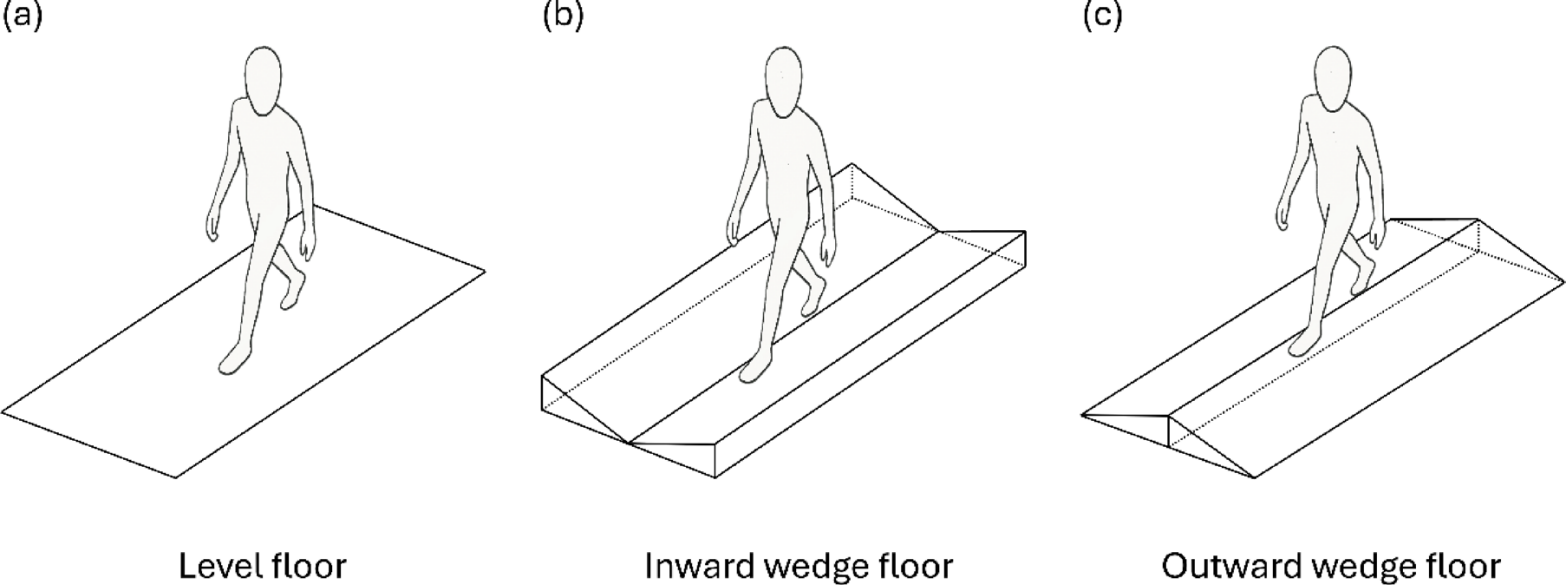
Walking surface conditions. Level floor (**a**), inward wedge (**b**), and outward wedge (**c**). Each platform measured 2.25 m in length, 0.35 m in width, 0.1 m in height. The wedge surfaces were inclined at 16°. Participants completed ten walking trials at a self-selected speed on each surface while kinematic data were recorded.

The participants then walked at a self-selected speed across a 3-m walkway under the following three different walking conditions: level floor, inward wedge, and outward wedge (Fig. 2). The bilateral wedges measured 2.25 m in length, 0.35 m in width, and 0.1 m in maximum height, which produced an inclination angle of 16°. Each participant completed 10 walking trials per condition in a randomized order, preceded by three practice trials. To minimize the potential fatigue effects between conditions, a 5-min rest period was provided. Ankle kinematics were simultaneously recorded using both the OMCs and IMUs. The sampling rates for the OMCs and IMUs were set to 200 and 400 Hz, respectively.

### Data processing

In this study, the shank and foot segments were modeled as rigid bodies, an assumption that does not explicitly account for soft tissue motion or deformation. Marker data from the OMCs were collected using Vicon Nexus software 2.16 https://www.vicon.com, Vicon Motion Systems Ltd., Oxford, UK) and processed based on the IOR foot model to estimate the 3D ankle kinematics across the anatomical planes. The captured trajectories were reconstructed and labeled using the IOR foot model. Static trials were performed to establish neutral joint positions, which served as a reference baseline for dynamic measurements. Joint angles were calculated using Python 3.10 (https://www.python.org), which applies anatomically aligned rotation matrices to enable precise analysis of 3D ankle kinematics in all three planes of motion^2,16^. The scripts were verified through internal testing, author code review, and peer validation.

IMU data were processed using MyoResearch software 3.18 (MR 3.18, https://www.noraxon.com, Noraxon, AZ, USA). Signals from the gyroscope, accelerometer, and magnetometer were fused using a quaternion-based algorithm integrated with an extended Kalman filter to reduce sensor noise and correct drift. This sensor fusion approach enhances the accuracy and reliability of ankle kinematic estimation by leveraging the complementary strength of each sensor to compensate for their individual limitations^19^. The Kalman filter operates under the assumption of linear error dynamics, which may not fully capture highly complex motion conditions. The Kalman filter refines the estimates by balancing measurement uncertainty with system dynamics^20^.

**Fig. 3:**
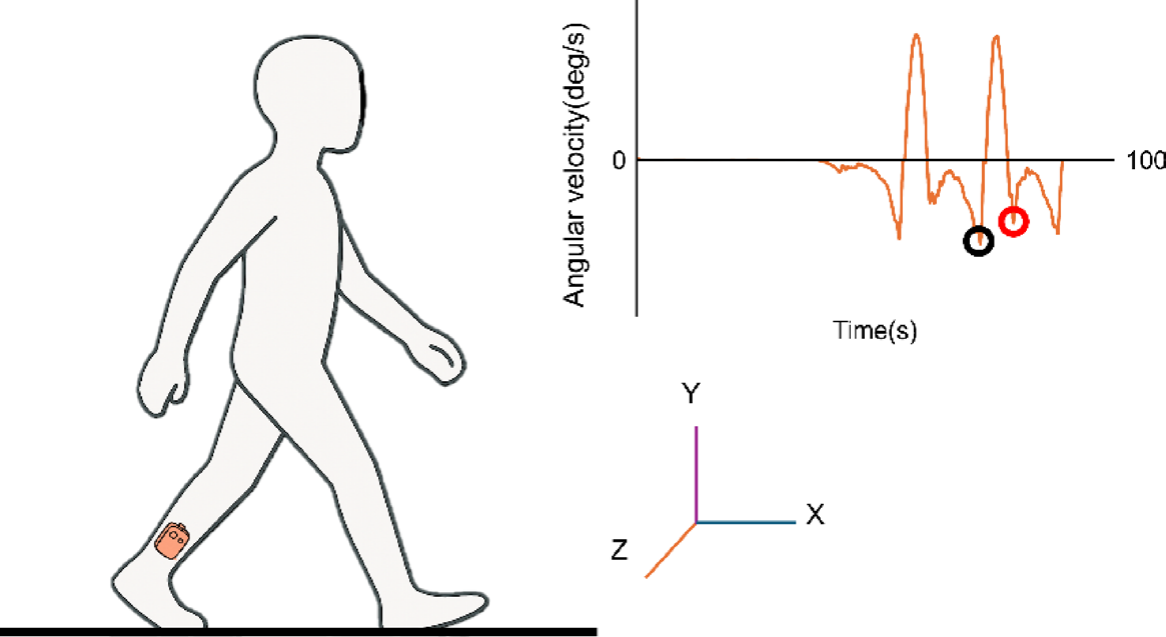
Gait event detection procedure. Heel strike (black circle) and toe off (red circle) events are detected from the Z-axis of angular velocity waveform of the shank mounted gyroscope.

Gait events were detected using the Z-axis angular velocity data from a gyroscope (Ultium EMG, Noraxon, AZ, USA) mounted on the shank (Fig. 3). This specific location is required to capture the signal patterns that indicate gait events. The gyroscope signals were smoothed using a median filter with a window size of 5 to reduce high-frequency noise. Heel strike and toe-off were identified in MR 3.18 based on the characteristic negative peaks in the Z-axis waveform^21^. The gyroscope data were recorded separately from the IMU system and synchronized with the IMU signals in MR 3.18. This allowed gait events to be directly aligned with the IMU timeline for precise identification during subsequent analyses.

**Fig. 4:**
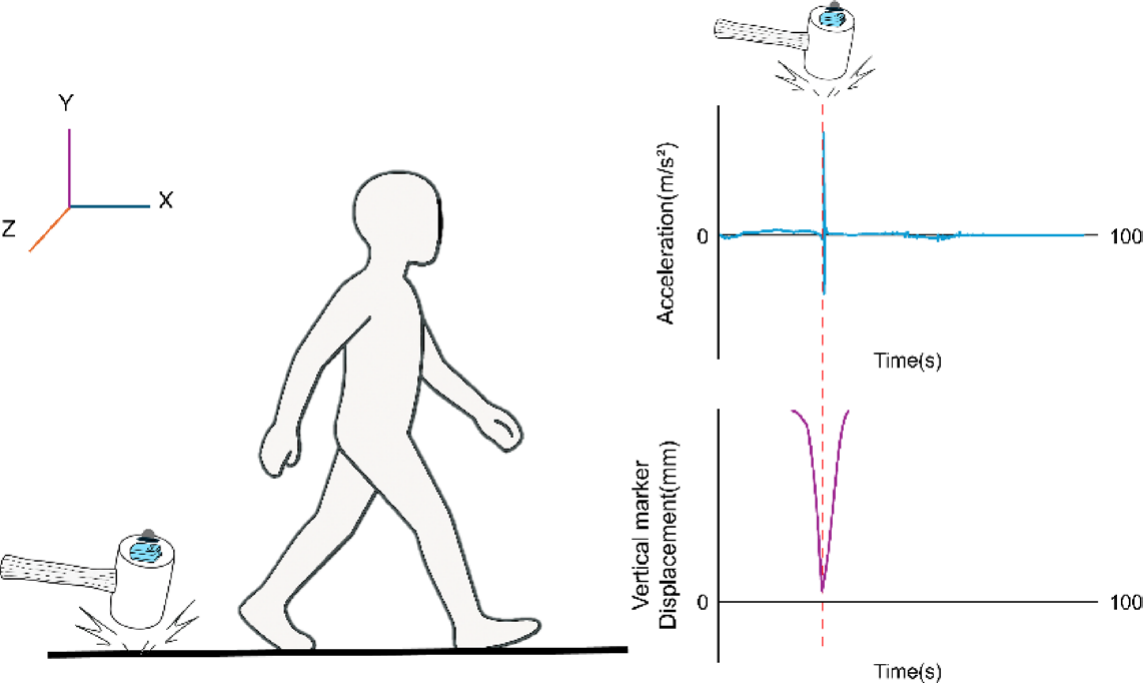
Synchronization procedure. A hammer strike generates a distinct acceleration peak in the accelerometer signal from the rubber hammer mounted EMG and a simultaneous vertical displacement in the Y-axis trajectory of the collocated optical marker. The corresponding timestamp (red dashed line) is used to synchronize data streams.

To synchronize the IMU system and OMCs, an accelerometer (Ultium EMG, Noraxon, AZ, USA) and an optical motion capture marker were attached to a rubber hammer. Striking the floor with the hammer generated a distinct peak in both the accelerometer signal and the OMCs marker trajectory. These peaks were used to align timestamps and synchronize the two measurement systems. ^22^ After synchronization, data from both systems were time-normalized to 100 frames per gait cycle to facilitate direct comparisons and account for temporal variations in gait (Fig. 4).

### Statistical analysis

The consistency between the IMU measurements and OMCs was evaluated using the coefficient of multiple correlation (CMC) across all three anatomical planes and walking conditions. CMC quantifies waveform similarity throughout the gait cycle, thereby offering a comprehensive assessment of kinematic patterns, as opposed to relying on discrete data points^23^. The values range from 0.00 to 1.00, with higher CMC scores indicating stronger agreement between systems. This method is particularly well-suited for assessing ankle kinematics under varying walking conditions as it captures the natural variability inherent in human gaits^24^.

Paired t-tests using one-dimensional statistical parametric mapping (SPM1d, https://spm1d.org) were performed using Python to evaluate the validity of the IMU-derived ankle kinematics via comparison with the OMCs measurements across all three planes. SPM1d was selected in this study owing to its capacity to identify specific intervals within the gait cycle that exhibit statistically significant differences, which enabled detailed comparisons of continuous kinematic data. In contrast to traditional discrete point analysis, SPM1d preserves the temporal structure of biomechanical signals and effectively controls Type I error across the entire time series^25^. This makes it particularly well-suited for gait analysis, wherein biomechanical events occur continuously throughout the movement cycle.

The root mean square deviation (RMSD) was also calculated to quantify the absolute differences in joint angle waveforms between IMU and OMCs measurements^6,9^. RMSD provides a measure of the average magnitude of error across the gait cycle, expressed in degrees. Lower values indicate closer correspondence between systems, allowing for an intuitive interpretation of deviation magnitude that complements correlation based and statistical analyses^26^.

The repeatability of the IMU measurements was assessed using the intraclass correlation coefficient (ICC), specifically the ICC (2,1) model, a two-way random-effects, single-measure form. This model accounts for both systematic and random errors, providing a conservative estimate of reliability across repeated trials for each walking condition. ICC values range from 0.00 to 1.00, with higher values indicating greater reliability. Values above 0.90 are interpreted as excellent, while values between 0.75 and 0.90 are considered good reliability and less than 0.5 are indicative of poor reliability^27–29^.

## Results

### Correlation of IMU and OMCs measurements

**Fig. 5:**
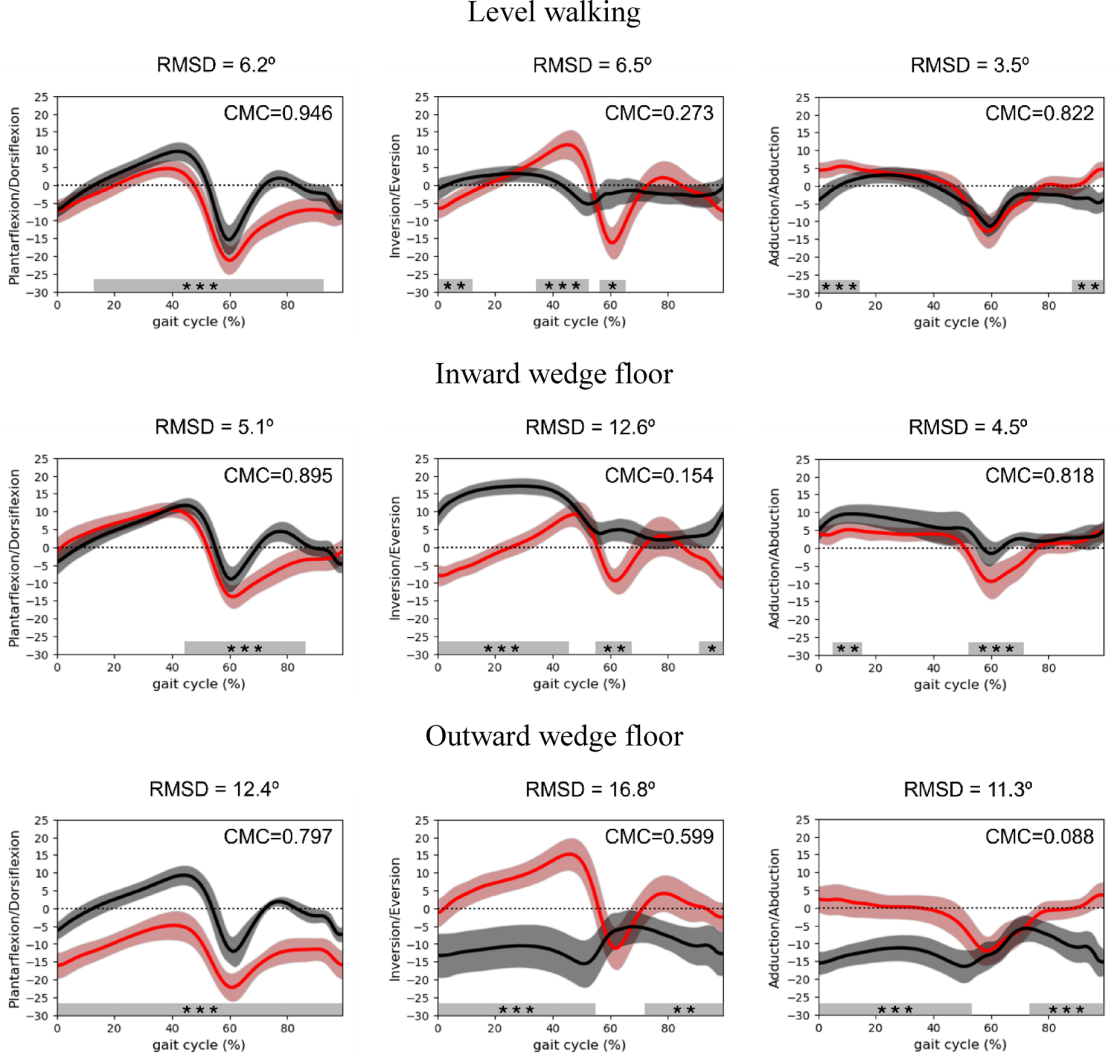
Comparison of 3D ankle kinematics between IMU and OMCs across walking conditions. Time series waveform of 3D ankle kinematics measured by the IMUs (red) and the OMCs (black), with shaded areas representing the standard deviation. Each panel displays the CMC and RMSD (°), which quantify waveform similarity and the magnitude of differences between the two systems. Gray bars beneath each plot indicate gait cycle intervals where SPM1d detected significant differences (*p < 0.05, **p < 0.01, ***p < 0.001).

The CMC was calculated to assess waveform similarities between the IMU and OMCs across the three anatomical planes (Fig. 5). Correlation strength varied with walking surface and plane of motion. In the sagittal plane, the IMU measurements demonstrated a strong agreement with the OMCs across all conditions. The highest correlation was observed during level walking (CMC = 0.946), indicating significant agreement between the systems. Under the inward wedge condition, correlation remained high (CMC = 0.895), suggesting sagittal plane kinematics were consistently captured despite altered walking surfaces. For the outward wedge condition, the correlation was slightly lower (CMC = 0.797) but reflected moderate agreement between the two measurement systems.

In the frontal plane, level walking demonstrated a relatively low correlation (CMC = 0.273), which indicated limited agreement between the two systems. This correlation decreased further under the inward wedge condition (CMC = 0.154) and highlighted substantial discrepancies in the measurements. Conversely, outward wedge walking showed an improved correlation (CMC = 0.599), which reflected moderate agreement. These findings suggest that IMU-derived measurements in the frontal plane are less consistent with OMCs compared to those in the sagittal plane, with particularly poor concordance observed during inward wedge walking.

In the transverse plane, level walking demonstrated a strong correlation (CMC = 0.822), indicating a high agreement between the measurement systems. This strong relationship was maintained under inward wedge conditions (CMC = 0.818), thereby suggesting effective transverse plane kinematics capture despite altered surface geometry. However, the correlation decreased significantly during outward wedge walking (CMC = 0.088), which indicated minimal agreement. This significant decrease suggests greater susceptibility of IMU-derived measurements to transverse plane errors under outward wedge conditions.

### Agreement between IMU and OMCs measurements

The SPM1d analysis revealed significant differences between IMU-derived and OMCs-based ankle kinematics at specific intervals of the gait cycle (Fig. 5). In the sagittal plane, significant deviations were observed with the IMUs during level walking, particularly throughout most of the stance and swing phases (14%–94%). In the frontal plane, notable differences were observed during the mid-stance (35%–52%) and early swing phases (56%–65%). Regarding the transverse plane, significant variations were noted during the initial contact (0%–5%) and terminal swing (89%–100%).

When walking on the inward wedge floor, the IMU recordings showed significant differences (*p* < 0.05) in the sagittal plane during terminal stance and throughout the swing phase (44%–86%). In the frontal plane, notable discrepancies were observed during the early stance (0%–45%), mid-swing (56%–67%), and terminal swing (91%–100%). In the transverse plane, significant deviations occurred during terminal stance and early swing phases (52%–72%).

During outward wedge floor walking, the IMU data showed significant divergence from the OMCs values across the entire gait cycle in the sagittal plane (0%–100%, *p* < 0.05). In the frontal plane, discrepancies were observed throughout the stance phase (0%–55%) and during terminal swing phase (73%–100%). Similarly, in the transverse plane, substantial deviations occurred during the stance (0%–53%) and terminal swing phases (75%–100%).

### Deviation between IMU and OMCs measurements

The RMSD analysis quantified the absolute magnitude of deviation in ankle kinematics between IMU-derived and OMCs-based measurements across walking conditions (Fig. 5). In the sagittal plane, RMSD values were relatively small during level (6.2°) and inward wedge (5.1°) walking, indicating close agreement between the two systems under these conditions. However, deviations increased substantially during outward wedge walking (12.4°).

In the frontal plane, RMSD values showed marked variability across conditions. Level walking presented differences (6.5°), whereas deviations were substantially larger on the inward wedge floor (12.6°) and reached their maximum on the outward wedge floor (16.8°).

In the transverse plane, RMSD values were lowest during level walking (3.5°) and slightly higher on the inward wedge (4.5°). In contrast, outward wedge walking demonstrated substantially larger deviations (11.3°).

### Repeatability of IMU measurements

**Table 1:** Intraclass correlation coefficients (ICC, model 2,1) for IMU-derived ankle kinematics.

ICC (95% CI)	Level walking	Inward wedge floor	Outward wedge floor
Sagittal plane	0.982 (0.977–0.987)	0.986 (0.987–0.990)	0.953 (0.938–0.965)
Frontal plane	0.976 (0.968–0.982)	0.961 (0.949–0.972)	0.933 (0.912–0.954)
Transverse plane	0.973 (0.965–0.980)	0.954 (0.940–0.966)	0.958 (0.945–0.969)

Mean ICC values and corresponding 95% confidence intervals (CI) are presented for each anatomical plane under the three walking surface conditions.

The repeatability of the IMU-derived ankle kinematics was assessed using ICC (2,1) across all walking conditions and anatomical planes (Table 1). During level walking, repeatability was the highest in the sagittal plane (ICC = 0.982), followed by the transverse plane (ICC = 0.976), whereas the frontal plane exhibited slightly lower but high reliability (ICC = 0.973).

Under inward wedge floor conditions, repeatability remained high in the sagittal (ICC = 0.986) and frontal (ICC = 0.961) planes, whereas the transverse plane showed a slight decrease (ICC = 0.954), thereby suggesting increased variability in movements.

Similarly, during outward wedge floor walking, repeatability remained relatively high in the sagittal (ICC = 0.953) and frontal (ICC = 0.933) planes, whereas the transverse plane (ICC = 0.958) demonstrated comparable reliability.

## Discussion

This study evaluated the agreement and repeatability of IMU-based 3D ankle kinematics relative to the gold standard OMCs across various walking conditions: level ground, inward wedge floor, and outward wedge floor. By examining all three anatomical planes, we assessed waveform similarity using CMC, continuous differences with SPM1d, absolute deviations with RMSD, and repeatability using the ICC. These analyses were conducted to determine whether a minimal IMU sensor configuration could provide ankle kinematic data as preliminary evidence toward preliminary applicability for functional interpretation outside the laboratory.

Under level walking conditions, the sagittal and transverse planes showed relatively high similarity between the IMU and OMCs measurements (CMC = 0.946 and CMC = 0.822, respectively), whereas the frontal plane exhibited a notably lower correlation (CMC = 0.273), indicating weak agreement in that plane. Despite the overall waveform similarity, statistically significant differences were observed in the sagittal plane between 14 and 94% of the gait cycle, in the frontal plane during mid-stance and early swing, and in the transverse plane during initial contact and terminal swing. RMSD values further supported these findings, with relatively small deviations in the sagittal (6.2°), frontal (6.5°) and transverse (3.5°) planes. Although these discrepancies were observed, repeatability remained high across all planes during level walking. These findings align with previous studies that suggest IMUs can provide a reasonable basis for functional interpretation of 3D ankle kinematics during level walking^3,9,29^. Furthermore, this study extends prior research by evaluating all three anatomical planes under diverse walking conditions, offering a more comprehensive framework for IMU assessment.

When participants walked on an inward wedge floor, the sagittal plane showed a relatively strong correlation (CMC = 0.895) with absolute deviations of 5.1°, despite significant differences of the gait cycle (44%–86%). This indicates that IMUs captured sagittal plane ankle motion with reasonable agreement under altered conditions. Conversely, the frontal plane demonstrated a much lower correlation (CMC = 0.154) and larger deviations (12.6°), with discrepancies during early stance, mid-swing, and terminal swing. These discrepancies likely reflect the increased inversion demands of the wedge surface, which represent a complex multisegmental motion that can amplify orientation misalignment when using only two IMUs. The transverse plane correlation (CMC = 0.818) was comparable to level walking, with small deviations (4.5°) limited to 52%–72% of the gait cycle. Despite these discrepancies, repeatability remained high (ICC > 0.940), providing preliminary evidence that minimal IMU setups could be applicable in the sagittal and transverse planes, while recognizing limitations in the frontal plane.

The most prominent discrepancies between IMU and OMCs measurements were observed under outward wedge floor conditions. Although the sagittal plane showed moderate correlation (CMC = 0.797) with significant differences throughout the gait cycle (0%–100%), with a deviation of 12.4°. The frontal plane showed moderate agreement (CMC = 0.599), with differences during 0%–55% and 73%–100% of the cycle, and an absolute deviation of 16.8°. Notably, the transverse plane correlation dropped substantially (CMC = 0.088), with significant differences during 0–53% and 75–100% and a deviation of 11.3°. This suggests that the rotational movements induced by the outward wedge floor may substantially reduce the agreement between the IMU and OMCs measurements. These discrepancies likely reflect the increased biomechanical complexity introduced by the outward wedge, especially eversion that affects IMU orientation estimation. Despite these limitations, repeatability remained high across all planes (ICC > 0.912), indicating that the IMU system provided internally consistent measurements. Although differences increased under complex conditions, measurements remained consistently reproducible across planes.

The consistent repeatability across walking conditions suggests potential for IMUs in gait monitoring beyond laboratory settings. Despite this potential, a notable observation across all walking conditions was the consistently lower agreement between the IMU and OMCs measurements in the frontal plane than in the sagittal and transverse planes. This discrepancy may be attributed to biomechanical complexity and methodological assumptions. Specifically, ankle inversion and eversion involve multisegmental interactions, particularly at the subtalar joint and midfoot, which become more pronounced under wedge walking^16,30,31^. However, IMUs, which are typically attached to the shank and dorsal foot, assume rigid-body behavior within each segment, overlooking inter-segmental dynamics^31,32^. In addition, while the OMCs defines axes anatomically, IMU axes are aligned functionally. This approximation may lead to misrepresentation of complex foot movements with the IMU coordinate system.

These differences were particularly pronounced during walking on an outward wedge floor. In this condition, depression of the lateral foot induces excessive inversion and increases the demand for supination. The complex interplay among the subtalar joint, midfoot, and shank during these motions may lead to kinematic misrepresentation by the IMUs. Moreover, the simplified sensor configuration permits only static calibration, which is typically performed in an initial standing posture, and assumes a neutral anatomical alignment. However, under wedged walking conditions, this assumption can result in a misalignment between the reference frame and dynamic ankle movements. Such a misalignment may lead to systematic offset errors that accumulate throughout the gait cycle^33,34^. Consequently, the agreement between the IMU-based measurements and OMCs was significantly reduced under this condition. The interaction between the biomechanical complexity and simplified sensor configuration could lead to the misrepresentation of 3D ankle kinematics by the IMU.

The observed discrepancies in the frontal plane highlight the need for advanced methodological approaches to improve the interpretation of multisegmental foot and ankle movements. Further research should explore functional calibration or postprocessing methods with minimal setups that can adapt to environmental changes. Moreover, methods are required that allow the interpretation of IMU-derived kinematics from a functional perspective, emphasizing movement patterns rather than exact anatomical replication. Finally, future studies should incorporate retest designs to establish reliability across sessions, which is critical for longitudinal analyses.

This study hypothesized that IMU-based measurements would demonstrate agreement with OMCs data across all three anatomical planes under varied walking conditions. With the sample restricted to healthy young adults, the findings cannot be readily generalized to clinical populations. Nevertheless, the high intra-session reliability observed suggests that, when interpreted from a functional perspective, IMU-derived kinematics may provide preliminary applicability as a minimal setup for capturing ankle motion outside the laboratory.

## Data Availability

The datasets generated and/or analysed during the current study are not publicly available due to institutional policies on participant privacy but are available from the corresponding author upon reasonable request.
